# Myopathic Ehlers-Danlos Syndrome (mEDS) Related to *COL12A1*: Two Novel Families and Literature Review

**DOI:** 10.3390/ijms26115387

**Published:** 2025-06-04

**Authors:** Luciano Merlini, Patrizia Sabatelli, Vittoria Cenni, Mariateresa Zanobio, Alberto Di Martino, Francesco Traina, Cesare Faldini, Vincenzo Nigro, Annalaura Torella

**Affiliations:** 1 Department of Biomedical and Neuromotor Science, University of Bologna, DIBINEM, 40136 Bologna, Italy; albertocorrado.dimartino@ior.it (A.D.M.); francesco.traina@ior.it (F.T.); cesare.faldini@unibo.it (C.F.); 2 CNR-Institute of Molecular Genetics “Luigi Luca Cavalli-Sforza”, Via di Barbiano 1/10, 40136 Bologna, Italy; sabatelli@area.bo.cnr.it (P.S.); vittoria.cenni@cnr.it (V.C.); 3 IRCCS, Istituto Ortopedico Rizzoli, Via di Barbiano 1/10, 40136 Bologna, Italy; 4 Department of Precision Medicine, University of Campania “Luigi Vanvitelli”, Via L. De Crecchio 7, 80138 Naples, Italy; mt.zanobio@gmail.com (M.Z.); vincenzo.nigro@unicampania.it (V.N.); 5 Ist Orthopedic Department, IRCCS Istituto Ortopedico Rizzoli, 40136 Bologna, Italy; 6 Ortopedia-Traumatologia e Chirurgia Protesica e dei Reimpianti d’anca e di Ginocchio, IRCCS Istituto Ortopedico Rizzoli, 40136 Bologna, Italy; 7 Telethon Institute of Genetics and Medicine, Via Campi Flegrei 34, 80078 Pozzuoli, Italy

**Keywords:** collagen type XII, *COL12A1*, collagen type VI, Ehlers-Danlos syndrome, UCMD2, BTHLM2, immunofluorescence, whole-exome sequencing (WES)

## Abstract

Myopathic Ehlers-Danlos syndrome (RmEDS) is an emerging hybrid phenotype that combines connective and muscle tissue abnormalities. It has been associated with variants of the *COL12A1* gene, which are known as Ullrich congenital muscular dystrophy-2 (UCMD2; 616470) and Bethlem myopathy-2 (BTHLM2; 616471). Here, we report two splicing mutations of *COL12A1* identified in three patients from two unrelated families who present a combination of joint hypermobility and axial, distal, and proximal weakness. The muscular strength of their neck and limb muscles was assessed at 4/5 (MRC); however, when measured with a myometer, the expected percentage by age and sex ranged from 35% to 40% for elbow flexion, 37% to 75% for knee extension, and was 50% for neck flexion. In addition to confirming the characteristic atrophy of the rectus femoris, we presented evidence of involvement of the neck and lumbar muscles through MRI and CT imaging. In vitro studies revealed filamentous disorganization and an altered pattern of collagen XII alpha 1 chain migration due to the skipping of exons 55 and 56 of collagen XII. Additionally, we review the myopathic involvement of COL12-RM in 30 patients across 18 families with dominant mutations and 15 patients from 13 families with recessive mutations.

## 1. Introduction

Collagen XII belongs to the family of fibril-associated collagens with interrupted triple helices (FACIT) [1,2,3,4], also including collagens IX, XIV, XVI, XIX, XXI, and XXII) and consists of a homotrimer of α1(XII) chains at the C-terminus. Collagen XII presents two collagenous-like 1 and 2 (CL1 and CL2, respectively) and three noncollagenous domains (NC1-NC3). Two distinct variants of collagen XII exist, arising from alternative mRNA splicing that differ in their NC3 domains [5,6]. It has been shown that collagen XII interacts with collagen I through the collagenous domain [7,8,9,10]. This binding regulates collagen I fibrillogenesis. The N-terminal globular domain 3, NC3, can interact with other molecules such as tenascin X [11], decorin, and fibromodulin [8,12]. Recent evidence has hypothesized a physical interaction between collagen XII and collagen VI; however, a direct or ternary structural interaction between these two collagens has not yet been reported [13]. Collagen XII is expressed in connective tissue, especially skin, tendon, ligaments, muscle, and bone, where it plays critical structural and functional roles. In particular, by influencing the organization of collagen fibrils, as well as participating in interfibrillar cross-linking [10,14,15,16], collagen XII contributes to the modulation of biomechanical properties of the extracellular matrix (ECM), with a crucial role in the regulation of tissue stiffness, elasticity, and tensile strength, with consequences on differentiation, proliferation, and cell migration. For these reasons, mutations in the collagen XII gene, *COL12A1*, may impair the structural properties of the ECM, negatively affecting its functions in response to mechanical, differentiative, or migratory stimuli.

Collagen XII-related myopathy (COL12-RM) is a novel, emerging hybrid phenotype that combines connective and muscle tissue abnormalities. It has recently been associated with variants of the *COL12A1* gene, which are referred to as myopathic Ehlers-Danlos syndrome (mEDS; OMIM 616470), Ullrich congenital muscular dystrophy-2 (UCMD2), and Bethlem myopathy-2 (BTHLM2; 616471). Currently, 31 families displaying this mixed phenotype have been described: 18 with autosomal dominant inheritance [17,18,19,20,21,22,23,24,25,26] and 13 with autosomal recessive inheritance [17,27,28,29,30,31,32] (Table 1 and Table 2). COL12-RM shares several aspects with the well-known collagen VI-related myopathy (COL6-RM) caused by dominant and recessive mutations in the three genes that encode collagen type VI (*COL6A1*, *COL6A2*, *COL6A3*). Both disorders present a clinical spectrum with varying severity, ranging from severe to mild, identified respectively by the UCMD and BM phenotypes. In COL6-RM, the criteria for distinguishing the two main phenotypes have been defined, and an intermediate severity form between the two has also been identified [33]. However, several factors have, thus far, hindered a clinically reliable distinction of the different phenotypes in COL12A1-RM: the limited number of patients, insufficient follow-up, and incomplete data regarding muscle strength, motor ability, and respiratory function, leading to evident negative repercussions on rehabilitative, orthopedic, and ventilatory treatment. This study aims to detail, in the two families, the mainly myopathic and functional aspects and to critically but constructively review the literature regarding the cases of myopathic Ehlers-Danlos syndrome (mEDS) related to *COL12A1*.

## 2. Results

### 2.1. Clinical and Laboratory Findings

#### 2.1.1. Family A

**Figure 1:**
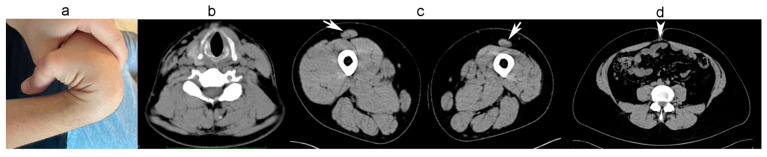
Clinical photo and muscle imaging of the 29-year-old patient from family A: (**a**) joint hypermobility; (**b**) hypotrophy of the right sternocleidomastoideus muscle (arrow); (**c**) round bilateral hypotrophy of rectus femoris muscle (arrows). Diffuse atrophy of the left thigh muscles after trochleoplasty performed 2 years before; (**d**) diastasis of the abdominal muscles (arrowhead), hypotrophy of the abdominal muscles, and partial fibro-adipose replacement of the inner lumbar paravertebral muscles.

In this family of non-consanguineous parents, both children, a boy and a girl, were affected. The mother, aged 51, had a normal physical examination. The father, aged 57 years, could not be examined. The first child was born at term by the Cesarian section. After birth, he was noted to have mild hypotonia, marked joint hypermobility, talus of the feet, and inguinal hernia. He walked at 17 months. Since childhood, he had difficulty running. At age 18 years, in another hospital, he underwent a needle muscle biopsy that showed a myopathic picture with increased endomysial connective tissue and fiber size variability but no necrosis. At age 27 years, he underwent trochleoplasty for recurrent left patella dislocation, trochlear dysplasia, and lateral discoid meniscus. A clinical examination at age 29 showed that he could walk on his toes and heels and climb stairs, but he could not run, mainly due to pain in his left knee. The examination showed blue sclerae, mild diffuse muscle hypotrophy, especially in the distal limbs and on the left side, and atrophy of the right sternocleidomastoid muscle (STCM). Muscle strength in the neck flexors and hand finger extensors measured 4/5 (MRC scale). At myometry, neck flexion measured 71 N (Newton) (50% predicted by age and sex), and right knee extension 209 N (75% expected). He scored 5 points on the Beighton Hypermobility Score (hands and right knee) (Figure 1a). Additionally, he had a mild finger contracture. His forced vital capacity was 90% of the predicted (he is a smoker). ECG and echocardiogram were normal. Creatine kinase was normal. Muscle CT showed atrophy of the right and hypotrophy of the left sternocleidomastoid muscles (STCM) (Figure 1b), diffuse hypotrophy of the left leg muscles, marked round atrophy of the left rectus femoris muscle (Figure 1b), mild diastasis of the abdominal muscles, and partial fibro-adipose replacement of the abdominal and lumbar paravertebral muscles.

The second child demonstrated normal motor development. At age 10, she began to report recurrent dislocations of her right knee, followed by frequent dislocations of her shoulders. Additionally, she experienced post-exercise myalgia and frequent muscle cramps. By age 14, she could walk on her toes but struggled to manage on her heels. A physical examination at age 14 revealed blue sclerae, mild diffuse muscle hypotrophy, and slight atrophy of the sternocleidomastoid and pectoralis muscles. Muscle strength for neck flexion and finger extension measured 4/5. She exhibited diffuse joint hypermobility with a Beighton score of 6/9. Myometry assessment indicated neck flexion measured 45 N (50% of the predicted value based on age and sex), hand grip strength was 30 kg bilaterally (normal), elbow flexion registered 79 N bilaterally (40% of the predicted), and knee extension was 103 N on the right and 125 N on the left (37% and 45% of predicted, respectively). Forced vital capacity and creatine kinase levels were normal. An echocardiogram revealed myxomatous mitral valve leaflets. An MRI of the thigh showed only mild muscle hypotrophy.

#### 2.1.2. Family B

**Figure 2:**
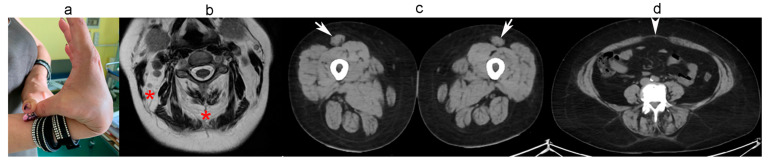
Clinical photo and muscle imaging of the 41-year-old patient from family B: (**a**) joint hypermobility; (**b**) MRI showing fibro adipose tissue between the markedly atrophic neck muscles (asterisks); (**c**) CT showing round bilateral hypotrophy of rectus femoris muscle (arrows). Left thigh muscles are more involved; (**d**) diastasis of the abdominal muscles (arrowhead), partial fibro-adipose replacement of abdominal and lumbar paravertebral muscles.

In this non-consanguineous family, the parents, aged 64 and 70, are unaffected, as are their sons, aged 33 and 28. However, their 41-year-old daughter was floppy as a child and began walking only after 18 months. As a child, she could not run like her peers and often fell. During childhood, she used to sit with her legs in a W shape or fully abducted to the side. Physical examination revealed moderate forearm and hand muscle hypotrophy, neck, hip, and knee flexors (4/5) weakness, difficulty walking on tiptoe, and diffuse joint hypermobility with a Beighton score of 6/9 (Figure 2a). At myometry, hand grip measured 18 kg on the right and 16 kg on the left (56% of predicted), elbow flexion 57 N on the right (35% of predicted). Forced vital capacity was 86% of predicted. Creatine kinase was normal. Muscle MRI showed increased fibro adipose tissue between the markedly atrophic neck muscles (Figure 2b). Muscle CT revealed, in addition to the characteristic round hypotrophy of the rectus femoris (Figure 2c), increased epimysial fat tissue of gastrocnemius muscles, diastasis of the abdominal muscles, and partial fibro-adipose replacement of the abdominal and lumbar paravertebral muscles (Figure 2d).

### 2.2. Mutation Analysis

**Figure 3:**
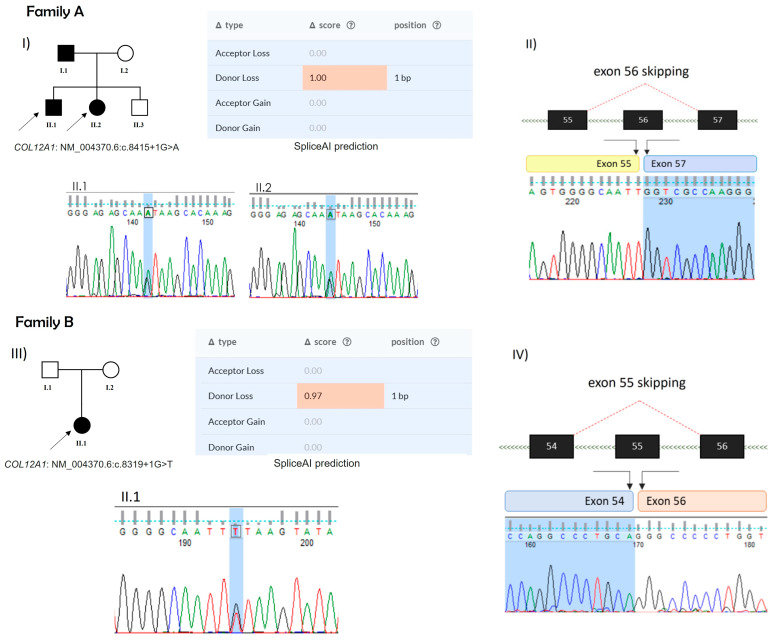
(**A**) Family pedigree, SpliceAI prediction, and Sanger sequence of genomic DNA variants for both probands (**I**); RNA analysis of II.1 proband, showing the skipping of exon 56 (**II**). (**B**) Family pedigree, SpliceAI prediction, and Sanger sequence of genomic DNA variants (**III**); RNA analysis showing the skipping of exon 55 (**IV**).

In family A, whole-exome sequencing (WES) data revealed the presence of the splicing variant, reported in ClinVar thanks to Solved-RD collaboration (VCV003256738.1), NM_004370.6: c.8415+1G>A in *COL12A1* gene, in both affected siblings. This variant was absent in gnomADv4.0 and classified as likely pathogenic according to ACMG guidelines (PVS1, PM2). It was also predicted to be deleterious by several bioinformatic tools, including SpliceAI, which predicted the loss of the donor splicing site with a maximum score of 1. The RNA analysis extracted from the muscle biopsy enabled us to confirm the skipping of exon 56 of *COL12A1*, resulting in an in-frame deletion in the COL2 domain of the protein (Figure 3A).

In family B, sequencing data performed on the affected proband revealed the presence of the splicing variant, NM_004370.6:c.8319+1G>T in the *COL12A1* gene. This variant, like that of family A, was not found in general population databases such as gnomADv4.0 and was classified as pathogenic according to ACMG guidelines (PVS1, PM2, PP5). In 2023, it was reported in ClinVar (VCV002506378.1) as pathogenic, and it was predicted to be deleterious by several bioinformatic tools. In particular, SpliceAI predicted that it could cause the loss of the donor splicing site with a score of 0.97. RNA analysis on muscular biopsy revealed the absence of the exon 55 in the *COL12A1* transcript, thus confirming the exon skipping as a consequence of the genomic variant. As with the skipping of exon 56 in Family A, the absence of exon 55 in Family B also resulted in an in-frame deletion in the COL2 domain of the protein (Figure 3B).

### 2.3. Immunofluorescence and Western Blot Analysis of Cell Cultures

**Figure 4:**
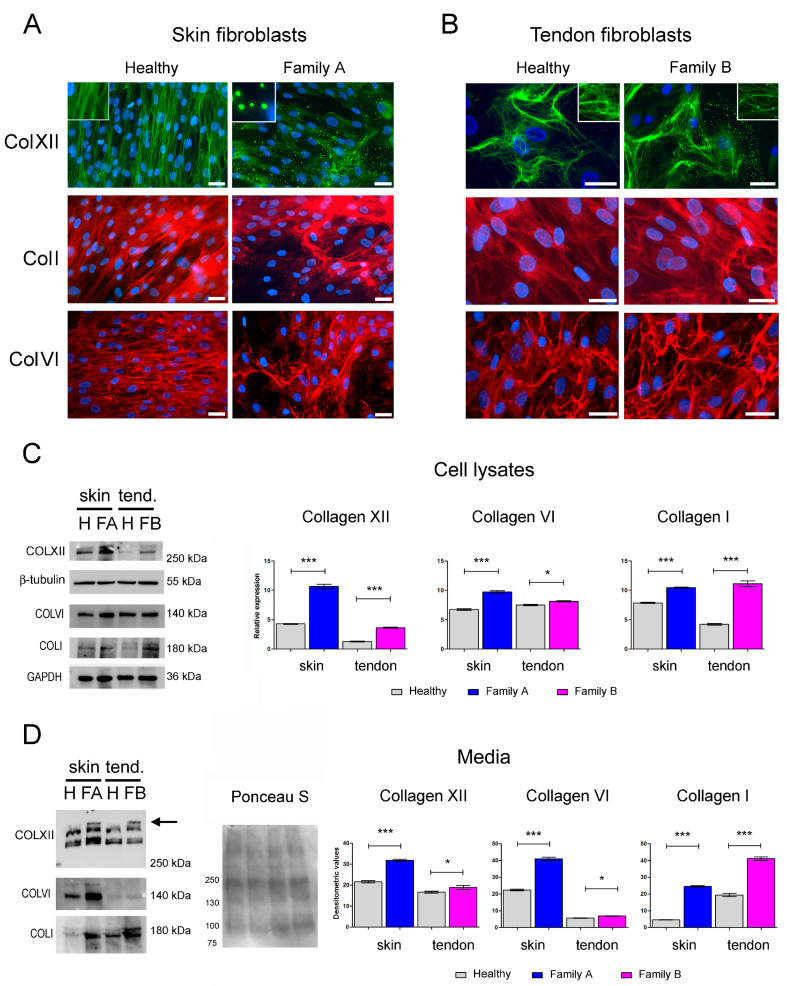
Effects of *COL12A1* variants on collagen XII organization and protein levels. Primary cultures from (**A**) skin and (**B**) tendon from a healthy subject (Healthy) and patients belonging to Family A and Family B were immunostained with antibodies anti-collagen XII (ColXII, green), collagen I and VI (ColI and ColVI, red). Magnified areas squared in white show details of collagen XII spot-like positive aggregates. Nuclei were counterstained with DAPI. Scale bar, 20 μm. (**C**,**D**) Primary cultures from skin and tendon biopsies from a healthy subject (H) and affected patients (Family A, FA, and Family B, FB) were lysed, and cultured media was collected. (**C**) Cell lysates were next assayed for collagen XII level of expression. In patients’ lysates, the amount of collagen XII was higher than that observed in controls. A similar behavior was observed also for collagen VI and I. Densitometric analysis shows relative expression of the proteins compared to loading controls: beta-tubulin (for collagen XII), and GAPDH (for collagen I and VI). (**D**) Analysis of culture media highlighted an increased amount of XII, VI, and I-types of collagen also in the media of pathological samples. A molecular weight shift (arrow) was evident in the collagen XII blot of pathological samples. Ponceau S staining is shown as the total protein loading control. Densitometric values are expressed in px^2^. * *p* ≤ 0.01; *** *p* ≤ 0.001.

To analyze the effect of both *COL12A1* variants on collagen XII protein synthesis and organization, primary cultured cells from a skin biopsy of the son of Family A and a tendon biopsy of the daughter of Family B were subjected to immunofluorescence and biochemical analyses. Immunofluorescence evaluation of the collagen XII alpha1 chain did not show cytoplasmic protein retention in either the controls or the patients’ cell cultures. However, the organization of the extracellular collagen XII network was altered in both patients. While in fibroblasts from the skin (Figure 4A) and tendon (Figure 4B) of unaffected individuals, collagen XII displayed a fibrillar pattern, the cells from patients showed a filamentous network associated with spot-like positive structures. These structures were prominent in Family A, common in Family B, and absent in normal skin and tendon cells. To determine if changes in the collagen XII network could affect the organization of other ECM components, collagen I, and collagen VI were subsequently evaluated. In skin fibroblasts from Family A, immunostaining of collagen I and collagen VI revealed a coarse organization of fibrils (Figure 4A). In contrast, collagen I and VI did not exhibit significant changes in the tendon fibroblasts of family B compared to normal tendon cultures (Figure 4B). Western blot analyses of cell lysates and culture media showed increased collagen XII expression in both patients’ cultures compared to the respective controls (Figure 4C,D). Specifically, biochemical analysis of culture media indicated abnormal migration patterns of secreted collagen XII. In pathological samples, a band likely corresponding to the long isoform of the monomeric α1(XII)-chain displayed a slower migration profile (Figure 4D). Interestingly, a significant increase in the expression of collagen I and VI was observed in the lysates and media of pathological samples compared to unaffected controls (Figure 4C,D).

## 3. Discussion

Our study confirms the clinical characteristics of mEDS, demonstrating combined joint hypermobility and neck, distal, and proximal weakness in three patients from two families. Diseases related to collagen VI and XII exhibit muscle biopsy characteristics that differ from those of muscular dystrophy; therefore, it is appropriate to collect the varied forms under the umbrella of related myopathy (RM), that is, COL6-RM and COL12A1-RM, respectively. The distinguishing features of dystrophic muscle biopsy include necrosis, regenerating fibers, and loss of muscle fibers replaced by connective tissue and fat. In UCMD1, muscle biopsy revealed a non-dystrophic myopathy [34] characterized by fiber size variation and prominent interstitial fibrosis disproportionate to the paucity of necrotic and regenerating fibers [35]. In UCMD2, muscle biopsy revealed a subtle variation in fiber size, consistent with a myopathic process [18,32] but without any overt signs of degeneration or regeneration [17].

Congenital or birth onset with hypotonia and contractures, along with delayed motor milestones, was reported in most cases of COL12A1-RM, including two of our three cases described here. Three patients had childhood onset [18,22], while the other two had adolescence onset [18], similar to one of our cases. All patients reported difficulty in running.

At the 166th ENMC International Workshop on Collagen VI Myopathies, it was proposed to restrict the UCMD label to patients who have never walked or have lost the ability to walk by age 12, the BTHLM label to patients who can walk during adulthood, and the intermediate label (INTM) to patients who lose ambulation during their teenage years [33]. Of the 33 patients with a dominant *COL12A1* mutation, the 31 who were able to walk, including the three patients we describe, were reported to have a BTHLM2 phenotype. However, only 14 of them were adults. Therefore, strictly speaking, 17 of the 31 should be classified as “currently BTHLM2 or Intermediate” depending on the outcome when they reach 18 yrs.

One patient (F4/P7), aged 8, was reported as INTM; she had global muscle weakness and significant scoliosis and could walk but fell frequently and needed support to climb stairs [19]. Another patient out of the 33 with a dominant mutation (F18/P30) showed a very severe UCMD2 phenotype [26]. This newborn presented with hypotonia, weak spontaneous movement, and respiratory failure at birth, along with unsuccessful attempts to wean the patient off the ventilator.

Of the 15 patients with recessive mutations, 10 were reported to have a severe UCMD2 phenotype: five never walked [17,28,31,32], one could take a few steps at age 4 [29], three were on NIV [32], and one aged 15 months was unable to sit [32]. Five of them instead were ambulant. One of these (F2/P3) had a definite BTHLM2 phenotype. He was born to consanguineous parents [27], had a congenital onset, walked at age 3, and by age 47, had normal proximal and distal manual muscle testing, and worked without physical concerns. Another patient with congenital hypotonia (F10/P11), at age 13, was able to run, jump, and climb stairs, exhibiting only mild proximal and axial weakness [32].

Of the 10 patients with the UCMD2 phenotype, muscle weakness was described as profound in three [17,28], proximal and distal in one [29], involving four limbs, and classified between 2/5 and 4+/5 (MRC) in five [30,33]. Of the 35 patients with a BHTLM2 phenotype, 10 had no information available on muscle strength. Among the remaining cases, three exhibited either proximal or distal weakness, while the others combined a mixed pattern that included axial or neck weakness in seven of them. The severity of proximal or distal weakness was described as mild, minimal, slight, or marked in six patients, while seven others were rated between 4/5 and 5-/5 (MRC). Neck weakness was defined as mild in two patients [1] or rated 2/5 and 4/5 in the other two [2].

Our new clinical data include a record of the strength of the neck and limb muscles measured with a myometer. Our patients’ proximal, distal, and neck muscle weakness was rated at 4/5. However, when strength was measured using the myometer, the percent predicted by age and sex varied from 37% to 75% for knee extension, 35% to 40% for elbow flexion, and 50% for neck flexion.

Hypermobility was reported as distal in 15 cases, generalized in 9, both distal and proximal in 10, marked in 1, and moderate in 1. The Beighton score was reported only in four patients, with scores ranging from 7 to 9 out of 9 [23,24], while in our patients, scores were between 5 and 6/9.

Only four BTHLM2 patients had a slight CK elevation [18]. Occasional CK elevation has been found in BTHLM1 without a clear correlation with disease onset or clinical severity [36].

Although the motor capacity of UCMD2 patients is similar to that of UCMD1, their respiratory function appears to be more severely compromised. Three out of 10 UCMD2 patients began non-invasive ventilation (NIV) before the age of 3 [17,31], while the need for NIV arises at the end of the first decade in UCMD1 [37,38], occurring only in two cases between the ages of 4 and 5.

Imaging of the rectus femoris muscle is typically recognized for differentiating patients affected by COL6-RM and COL12A1-RM. In patients with the collagen VI mutation, the central part of the muscle appears preserved, while the peripheral part adjacent to the fascia is replaced by fibro-adipose tissue [39,40,41,42]. This suggests that the peripheral muscle fibers in contact with the muscle fascia degenerate earlier than the central fibers, which stay intact for longer. We have previously shown in COL6-RM that the muscle biopsy and deep fascia of the rectus femoris muscle exhibit a similar distinct pattern of outside–in muscle degeneration followed by fat substitution, as evidenced by muscle imaging [43]. An early loss of fast-twitch fibers and significant ectopic fatty infiltration characterizes the changes at the interface between the muscle and the epimysium. We suspected that, in COL6-RM, the pathogenic mechanism is that peripheral muscle fibers, which endure a heavier workload and greater energy demands, may experience earlier effects from mitochondrial dysfunction associated with defective autophagy [43]. On the contrary, in patients with the *COL12A1* mutation, the muscle is variably reduced in size [44] and surrounded by an increased thickness of connective tissue (epimysium), as though the muscle is being choked by the excessive connective tissue surrounding it [18,20,22,25,31,32].

Our findings demonstrate that the *COL12A1* variants presented in this study cause structural and biochemical changes in collagen XII within cell cultures derived from biopsies of affected patients. Consistent with published evidence regarding similar *COL12A1* deletions [23], the mutations reported here not only disrupt the organization of collagen XII in the ECM but also affect its protein level and post-translational modifications. The identified mutations are expected to affect the CL2 domain of the collagen XII α1 chain, which regulates the interaction between collagen XII and collagen I and plays a role in collagen fibril assembly. In line with the established influence of FACIT collagens on modulating fibrillogenesis [44] and the recognized function of the CL2 domain in facilitating collagen I fibril formation, we observed that the *COL12A1Δ56* mutation (Family A) also affected the organization and pattern of collagen I secretion, as indicated by the coarse arrangement of collagen I fibrils in the ECM and the increased amount of this protein released into the culture medium. These findings suggest that the *COL12A1Δ56* mutation disrupts collagen XII’s ability to form stable interactions with ECM partners. Interestingly, in pathological cells, collagen VI also showed alterations similar to those of collagen I. In this regard, it is important to note that although a direct interaction between collagen XII and collagen VI has not been reported, several data point to a functional interaction between these two collagens [13]. For instance, as previously mentioned, patients with *COL12A1* mutations show clinical features similar to those of COL6-RM patients; furthermore, the expression and organization of collagen XII are altered in tendon cell cultures from COL6-RM [45,46]. Although less evident, the *COL12A1Δ55* mutation (Family B), which results in the complete removal of exon 55, influenced the organization and amount of collagen XII released into the medium of cultured tendon cells. Additionally, collagen I and collagen VI appeared altered, as indicated by their increased amount in cell lysates and media. In contrast, the organization of collagen I and VI in this patient was not significantly altered, as shown by immunofluorescence analysis. Future investigations will be essential to determine whether the effects of the *COL12A1Δ56* mutation on ECM organization are specific to this mutation (deletion of exon 56) or if they represent a unique characteristic of skin compared to tendons.

## 4. Materials and Methods

### 4.1. Patients and Methods

All subjects gave their informed consent before participating in the study. The study was conducted following the Declaration of Helsinki, and the protocol was approved by the Ethics Committee at the Rizzoli Orthopedic Institute (project identification code PG0006743, approval date: 5 July 2017).

A handheld dynamometer (CT 3001, C.I.T. Technics, Groningen, The Netherlands) was used to measure the muscle strength of elbow flexion and knee extension in newtons. Only the highest score obtained on either side was used and reported as % of predicted by sex and age [47,48,49]. A Jamar dynamometer (J A Preston Corporation, New York, NY, USA) was used to measure grip strength in kilograms. Hand-grip strength was assessed in a seated position, with the tester supporting the weight of the dynamometer by resting it on their palm. The highest score recorded on either side was reported as a percentage of the predicted values by sex and age (Jamar manual).

Primary human cultures of tendon and skin were obtained from bioptic samples of affected individuals and two age-matched volunteer controls who underwent elective surgery. Control skin fibroblast cultures were derived from a biopsy of a 37-year-old male subjected to rotator cuff surgery, while control tendon fibroblasts were obtained from a small fragment of the semitendinosus tendon of a 25-year-old female subjected to autologous graft for ACL reconstruction. Harvested skin and tendon of controls used in this study were not involved in any known pathological process.

To obtain primary cultures, tendon fragments or skin biopsies were subjected to mechanical dissociation. Tendon cells were grown in Dulbecco’s Modified Eagle Medium (DMEM)-Glutamax supplemented with 1% antibiotics and 10% fetal bovine serum (FBS) [45], while dermal fibroblasts were cultured in DMEM-Glutamax enriched with 20% FBS and 1% antibiotics. Cells were maintained in a humidified atmosphere with 5% CO_2_ at 37 °C.

### 4.2. Genetic Analysis

According to the manufacturer’s instructions, genomic DNA was extracted from fresh peripheral blood samples using FlexiGene DNA Kit (Qiagen, Hilden, Germany). Library preparation has been performed using SureSelectQXT Automated Target Enrichment for the Illumina Platform, enriched with the SureSelect Human All Exon v8 (Agilent Technologies, Santa Clara, CA, USA), following the manufacturer’s instructions. The obtained libraries were assessed for quantity using Qubit dsDNA HS Assay Kit for Qubit 3.0 fluorometer (Thermo Fisher, Waltham, MA, USA) and quality using the High Sensitivity DNA ScreenTape Assay kit for Agilent 4200 TapeStation (Agilent Technologies, Santa Clara, CA, USA). Libraries were then sequenced using the high throughput Illumina NovaSeq 6000 system, performing paired-end runs covering at least 2 × 150 nt. (Illumina Inc., San Diego, CA, USA). A medium coverage of 100X is usually obtained, with 90% of the regions covered by at least 40 reads. The generated sequences were analyzed using an in-house pipeline designed to automate the analysis workflow [50]. Direct Sanger sequencing was performed to validate the pathogenic variants using the BigDye version 3.1 sequencing kit on a 3500xl Genetic Analyzer. (Applied Biosystems, Massachusetts, USA). Total RNA was extracted from the patient’s muscle biopsy using TRIzol RNA isolation reagents (Thermofisher Scientific, Waltham, MA, USA) according to the manufacturer’s specifications. According to the manufacturer’s instructions, RNA was retrotranscribed using SuperScript III RT (Invitrogen, Carlsbad, CA, USA) and random primers. Complementary DNA (cDNA) was then used to amplify the *COL12A1* fragment spanning exons 52–61 for both probands. The PCR product was subsequently analyzed by bidirectional direct Sanger sequencing, as previously described.

### 4.3. Immunofluorescence of Dermal and Tendon Fibroblast Culture

Immunofluorescence and Western blot analyses were performed on cultured fibroblasts obtained from a skin biopsy of Family A and a tendon biopsy of Family B, and these were compared with age-matched normal skin and tendon cultures. Skin and tendon fibroblast cultures were obtained following well-established protocols [51,52]. For immunofluorescence analysis, cells were grown on coverslips, treated for 24 h with 0.25 mM L-ascorbic acid post confluence, and incubated with antibodies against collagen XII (Santa Cruz Biotechnology Inc., Santa Cruz, CA, USA), collagen VI, and collagen I (Millipore), along with FITC (Fluorescein isothiocyanate) or TRITC (Tetramethylrhodamine) conjugated anti-mouse or anti-rabbit secondary antibodies (DAKO). Cell nuclei were stained with 1 mg/mL DAPI (4′,6-diamidino-2-phenylindole) (Sigma-Aldrich, St Louis, MO, USA). Samples were mounted with an anti-fading reagent (Invitrogen, Carlsbad, CA, USA) and observed with a Nikon epifluorescence microscope.

### 4.4. Western Blotting

For Western blot analysis, cultured cells were washed in PBS and scraped into SDS-lysis buffer. A total of 10–20 µg of lysates was resolved on a 4–15% precast polyacrylamide gel (Bio-Rad Laboratories SrL Italy, Segrate, Italy), transferred to nitrocellulose membranes (Santa Cruz Biotechnology, DBA SrL Italy, Segrate, Italy), and immunoblotted with anti-collagen XII (Clone A-11), collagen VI, and collagen I antibodies (all from Santa Cruz Biotechnologies). At the same time, GAPDH (Merck Millipore, St. Louis, MO, USA) served as a loading control. Equal loading control of cell culture media was revealed by Ponceau S staining (Thermofisher Scientific, Waltham, MA, USA). Chemiluminescence images were captured using the ChemiDoc MP Imaging System (Bio-Rad, Hercules, CA, USA). Densitometric analyses were conducted with ImageJ software, version 1. Data are presented as the mean ± SD of three biological replicates.

### 4.5. Statistical Analysis

Statistical significance was determined using Student’s *t*-test. Statistical analyses were performed with GraphPad Prism version 5.0 for Windows (GraphPad Software, La Jolla, CA, USA). Results were considered statistically significant for *p*-values less than 0.05.

## Figures and Tables

**Table 1:** Clinical, laboratory, and genetic data of the families with dominant *COL12A1* mutation.

Family/ Patient	Gender	Onset	Age (years)	Able to Walk	FVC% Predicted	NIV Age (Years)	Muscle Weakness (MRC)	CK (U/L)	Hypermobility (BS)	*COL12A1* Variant #	Ref.
F1/P1	M	Birth	3	y	-	-	-	-	D	c.7001T>C, p.Ile2334Thr	[17]
F2/P2	F	Childhood	42	y	Normal	-	Mild P and N	93	Generalized	c.8357G>A, p.Gly2786Asp	[18]
F2/P3	F	Birth	6	y	Normal	-	Mild N, min P	747	Generalized	Same family variant	
F3/P4	M	Childhood	48	y	Normal	-	P	973	-	c.5893Arg, p.Arg1965Cys	
F3/P5	M	Adolescence	22	y	Normal	-	P	1310	n	Same family variant	
F3/P6	M	Adolescence	27	y	79%	-	P	824	n	Same family variant	
F4/P7	F	Birth	8	y	-	-	Global	Normal	Marked	c.8329G>C, p.Gly2777Arg	[19]
F5/P8	M	Birth	15	y	58%	-	A-P and D (4+)	Normal	Generalized	c.8100+2T>C	[20]
F5/P9	M	Birth	12	y	91%	-	A and P (5-), D (4)	-	Generalized	Same family variant	
F5/P10	F	Birth	40	y	105%	-	A-P and D (4+)	-	Generalized	Same family variant	
F5/P11	F	-	76	y	58%	-	P (5-), D (4-)	-	D	Same family variant	
F6/P12	F	Birth	4	y	-	-	-	-	D	c.7196G>A, p.Gly2399Glu	[21]
F6/P13	M	-	34	y	-	-	-	-	D	Same family variant	
F6/P14	M	-	34	y	-	-	-	-	D	Same family variant	
F7/P15	M	Birth	5	y	95%	-	A (2/5), P and D (4)	174	D and P	c.7951-630_8100+991del1771ins10	[22]
F7/P16	F	Birth	37	y	104%	-	D (4+)	60	D and P	Same family variant	
F7/P17	M	Birth	33	y	96%	-	D (4)	101	D and P	Same family variant	
F8/P18	M	Childhood	62	y	115%	-	D (4)	106	n	c.8276G>A, p.Gly2759Asp	
F9/P19	M	Birth	4	y	81%	-	Mild P and D	103	D and P	c.8453G>A, p.Gly2818Glu	
F10/P20	M	Birth	3	y	-	-	A (2/5), mild P and D	99	D and P	c.8065G>A, p.Gly2689Arg	
F11/P21	M	Birth	4	-	-	-	P	198	Generalized	c.8415+1_8415+10del	[23]
F12/P22	F	Birth	4	y	-	-	-	258	Generalized (7/9)	c.8265+1G>A	
F13/P23	F	Birth	13	y	-	-	-	73	Generalized (9/9)	c.8178+3A>C	
F13/P24	F	Birth	10	y	-	-	-	-	Generalized (9/9)	Same family variant	
F13/P25	M	Birth	39	y	-	-	-	254	Moderate	Same family variant	
F14/P26	M	Birth	7	y	-	-	-	-	D	c.8100+3_8100+6delGAGT	
F15/P27	M	Birth	18	y	-	-	-	146	-	c.5587C>T, p.Arg1863Cys	
F16/P28	F	-	14	y	-	-	Upper limbs (4/5)	42	Generalized (7/9)	c.8336G>A, p.Arg2779His	[24]
F17/P29	F	Birth	8	y	73	-	Shoulder, marked	-	-	In-frame deletion of exons 45–54	[25]
F18/P30	F	Birth	0.1	-	-	0.1	-	380	-	c.7622C>T, p.Ser2541Phe	[26]
F19/P31	M	Birth	29	+	90	-	A and D (4/5)	Normal	D (5/9)	c.8415+1G>A	This report
F19/P32	F	Adolescence	14	+	Normal	-	A and D (4/5)	Normal	Generalized (6/9)	Same family variant	
F20/P33	F	Birth	41	+	86	-	N, hip, knee (4/5)	Normal	Generalized (6/9)	c.8319+1G>T	

P2 is P3’s mother, P4 is the father of P5 and P6, P8 is P9’s brother, P10 is the mother of P8 and P9, P11 is P10’s mother, P12 is the daughter of P13, P14 is the monozygotic twin of P13, P15 is P16’ son, P17 is P16’s brother, P23 and P24 are daughters of P25, P31 is P32’s brother; FVC% = forced vital capacity, percent predicted; NIV = non-invasive ventilation; MRC = Medical Research Council; CK = creatine kinase; BS = Beighton score; M = male; F = female; y = yes; n = no; - = not available; A = axial; N = neck; P = proximal; D = distal; **# =** The variants shown are described using the NM_004370.6, and all variants are in heterozygous status.

**Table 2:** Clinical, laboratory, and genetic data of the families with recessive *COL12A1* mutation.

Family/ Patient	Gender	Onset	Age (Years)	Able to Walk	NIV Age (Years)	CK (U/L)	Muscle Weakness (MRC)	Hypermobility (BS)	COL12A1 Variant:	Status	Ref.
F1/P1	M	Birth	9	Never	<3	Normal	Profound, mild facial	D	c.8006+1G>A	Hom	[17]
F1/P2	M	-	-	Never	n	Normal	Profound	D	Same family variant	Hom	
F2/P3	M	Birth	47	y	n	93	P and D	D	c.395-1G>A	Hom	[27]
F3/P4	M	Birth	6	Never	-	-	Profound	-	c.8828C>T, p.(Pro2943Leu)	Hom	[28]
F4/P5	F	-	4	Few steps	-	90	P and D	D	c.7541A>G, c.8369C>T	CompHet	[29]
F5/P6	F	-	3	y	n	Normal	Four Limbs (4/5)	D	c.8903C>T, p.Pro2968Leu	Hom	[30]
F6/P7	F	Birth	1.9	Never	<2	51	-	D	c.4240C>T, p.R1414X	Hom	[31]
F7/P8	F	Congenital	1.25	n	n	-	ULs (3/5), LLs (2/5)	D and P	c.57904+2T>A; c.5269C>T	CompHet	[32]
F8/P9	F	Congenital	18	n	y	-	Four Limbs (4/5), N (2/5)	D and P	c.8464C>T	Hom	
F9/P10	M	Congenital	4	-	y	-	Generalized	D	c.6340+1G>TinsA	Hom	
F10/P11	M	Congenital	13	y, run	y	-	Mild P and A	D	c.946_947insA	Hom	
F11/P12	F	Congenital	3	y	n	-	Four Limbs (4/5),N(2/5)	D and P	c.3329C>A, c.4177del	CompHet	
F11/P13	M	Congenital	1.25	y	n	-	Four Limbs (3/5)	D and P	Same family variant	CompHet	
F12/P14	M	Congenital	3	n	y	-	ULs and LLs (3/5)	Hands and feet	c.5664+1G>A; c.6127delG	CompHet	
F13/P15	M	Congenital	16	Never	y	-	D > P, LLs > ULs	D and P	c.5230+1G>A	Hom	

P1 is the P2’s brother; NIV = non-invasive ventilation; MRC = Medical Research Council; CK = creatine kinase; BS = Beighton score; Ref. = reference; M = male; F = female; y = yes; n = no; - = not available; A = axial; N = neck; P = proximal; D = distal; ULs = upper limbs; LLs = lower limbs; X = stop codon.

## Data Availability

The original contributions presented in this study are included in the article. Further inquiries can be directed to the corresponding authors.
